# Newcastle disease virus NP and P proteins induce autophagy via the endoplasmic reticulum stress-related unfolded protein response

**DOI:** 10.1038/srep24721

**Published:** 2016-04-21

**Authors:** Jing-Hua Cheng, Ying-Jie Sun, Fan-Qing Zhang, Xiao-Rong Zhang, Xv-Sheng Qiu, Li-Ping Yu, Yan-Tao Wu, Chan Ding

**Affiliations:** 1 Department of Avian Infectious Diseases, Shanghai Veterinary Research Institute, Chinese Academy of Agricultural Sciences, Shanghai, 200241, P.R. China; 2 Jiangsu Co-Innovation Center for Prevention of Animal Infectious Diseases and Zoonosis, College of Veterinary Medicine, Yangzhou University, Yangzhou, 225000, P.R. China; 3 College of Agriculture and Biology, Shanghai Jiaotong University, Shanghai, 200240, P.R. China

## Abstract

Newcastle disease virus (NDV) can replicate and trigger autophagy in human tumor cells. Our previous study confirmed the critical role of autophagy in NDV infection. Here we studied the role of NDV structural proteins in the induction of autophagy through endoplasmic reticulum (ER) stress-related unfolded protein response (UPR) pathways. Ectopic expression of the NDV nucleocapsid protein (NP) or phosphoprotein (P) was sufficient to induce autophagy. NP or P expression also altered ER homeostasis. The PERK and ATF6 pathways, but not the XBP1 pathway, all of which are components of the UPR, were activated in both NDV-infected and NP or P-transfected cells. Knockdown of PERK or ATF6 inhibited NDV-induced autophagy and reduced the extent of NDV replication. Collectively, these data suggest not only roles for the NDV NP and P proteins in autophagy, but also offer new insights into the mechanisms of NDV-induced autophagy through activation of the ER stress-related UPR pathway.

Newcastle disease virus (NDV) is a single-stranded, non-segmented, negative-sense, enveloped RNA virus that belongs to the genus *Avulavirus* in the family *Paramyxoviridae*. The ~15.2 kb NDV genome encodes six major structural proteins, including the nucleocapsid protein (NP), phosphoprotein (P), matrix protein (M), fusion protein (F), hemagglutinin-neuraminidase protein (HN), and the large polymerase protein (L)^1^,^2^. Although NDV is an avian pathogen, its effect on human cancers has been widely reported^3^,^4^. NDV selectively replicates in and destroys human tumor cells without affecting normal cells and thus provides a potential therapeutic approach for treating human cancers^5^,^6^.

Autophagy is an evolutionarily conserved intracellular process that generates a double-membrane vesicle to deliver portions of the cytoplasm to the lysosome for digestion and recycling^7^,^8^. Autophagy can be initiated in response to diverse stress stimuli, including nutrient deficiency, endoplasmic reticulum (ER) stress, oxidative stress, and virus infection^8^. During autophagy, Microtubule associated protein 1 light chain 3 (LC3) undergoes a posttranslational modification to form lipidated LC3-II that serves as an autophagosomal marker in host cells. A multifunctional protein p62 (SQSTM1) interacts with LC3-II and serves as a substrate for autophagic degradation^9^. There are several known pathways leading to autophagy, such as the serine/threonine kinase mammalian target of rapamycin (mTOR) pathway and phosphatidylinositol 3-kinase (PI3K) signaling pathway which regulate autophagy negatively or positively^10^. The autophagy-related protein Beclin-1 (homologue of yeast Atg6) is critical for the signaling pathways, and its increased expression suggests increased cellular autophagic activity^11^,^12^.

Many recent studies have focused on the role of autophagy in viral infection^13^,^14^. Autophagy may provide an intrinsic host defense mechanism against invading viruses^15^. For example, studies of Herpes simplex 1 (HSV1), vesicular stomatitis (VSV), and Sindbis viruses indicated that they are susceptible to autophagy, and inhibition of autophagy leads to increasing their replication and virulence^16^,^17^,^18^. However, the role of autophagy in virus infection is complicated, because some viruses manipulate autophagy for their own benefit. NDV, Classical swine fever virus, and porcine reproductive and respiratory syndrome virus exploit autophagic vesicles to facilitate their own replication^14^,^19^,^20^.

The endoplasmic reticulum (ER) is a multifunctional organelle involved in post-translational modifications, folding, and oligomerization of newly synthesized proteins. However, several endogenous imbalances in cells can contribute to an ER malfunction known as ER stress^21^,^22^. To maintain ER homeostasis, cells have evolved an adaptive response known as the unfolded protein response (UPR) pathway, which refolds or degrades unfolded/misfolded proteins to maintain ER homeostasis. The UPR is characterized by the enhanced expression of several chaperones, such as GRP78/BiP, GRP94, calreticulin, and proteins involved in the disulfide bond formation^22^,^23^. For example, the encephalomyocarditis virus 2C and 3D proteins trigger the up-regulation of GRP78, GRP94, and calreticulin during ER stress, an increase in GRP78 protein levels was observed in Dengue virus and West Nile virus induced ER stress^21^,^24^,^25^. The UPR can be mediated by the activation of three distinct stress transducers including inositol-requiring enzyme 1 (IRE1), activating transcription factor 6 (ATF6), and PKR-like ER protein kinase (PERK), which sense the level of unfolded proteins in the ER lumen^22^,^26^. In response to the ER stress, activated PERK phosphorylates the eukaryotic initiation factor eIF2α thus resulting in translation attenuation, ATF6 translocates from the ER to the Golgi apparatus and is cleaved by trans-membrane proteases, activated ERN1 splices 26 nucleotides from the XBP1 (X-box binding protein 1) mRNA to create a spliced XBP1 mRNA which allows expression of the full-length transcription factor XBP1^27^,^28^.

Some viruses have been demonstrated to induce ER stress and trigger UPR pathway in infected cells. ER stress can trigger autophagy through activation of UPR components^29^,^30^,^31^. Our previous studies demonstrated that NDV could induce autophagy in both cultured cells and in chicken models to benefit its replication^20^,^32^. However, the mechanisms by which NDV proteins induce autophagy are unknown. Furthermore, whether the autophagy induced by NDV infection is associated with the UPR has not been elucidated yet. Here we show that the NDV P and NP proteins trigger autophagy in A549 cells. Ectopic expression of P and NP altered ER homeostasis through the upregulation of the ER stress marker proteins GRP78 and GRP94. Finally, we demonstrated that NDV P and NP protein-induced autophagy is regulated through the PERK and ATF6 pathways and knockdown of these proteins suppresses NDV replication.

## Materials and Methods

### Cells, virus and plasmids

The human non-small-cell lung cancer (NSCLC) cell line A549 was purchased from ATCC (Manassas, VA, USA) and cultured in Dulbecco’s modified Eagle’s medium (DMEM)(GIBCO)(Grand Island, NY, USA) supplemented with 10% fetal bovine serum (Invitrogen, Carlsbad, CA, USA) at 37 °C humidified atmosphere containing 5% CO_2_.

The NDV velogenic strain Herts/33 and the lentogenic strain La Sota were obtained from the Chinese Institute of Veterinary Drug Control (IVDC) (Beijing, China). Viral titers were determined by using the Reed–Münch method and were expressed as the tissue culture infective dose 50 (TCID_50_) per milliliter. Plasmid p3 × FLAG-CMV-14 was purchased from Sigma-Aldrich (St. Louis, MO, USA). Plasmid pEGFP-LC3 was purchased from Origene (Rockville, MD, USA). Plasmids pGL3-Basic and pRL-TK were purchased from Promega (Madison, WI, USA).

### Antibodies and reagents

Thapsigargin (Tg), mouse monoclonal anti-Flag M2 antibody, rabbit polyclonal anti-LC3B antibody, rabbit polyclonal anti-p62/SQSTM1 antibody, and mouse monoclonal anti-actin antibody were purchased from Sigma-Aldrich. Mouse monoclonal antibodies against GRP78, GRP94, ATF6, ATF4, Beclin-1, PERK, phospho-PERK, eIF2α, and phospho-eIF2α were purchased from Cell Signaling Technology (Danvers, MA, USA). Rabbit polyclonal IRE 1 and phospho-IRE 1 were purchased from Abcam (Cambridge, MA, USA). Horseradish peroxidase (HRP)-conjugated goat anti-rabbit or -mouse secondary antibody was purchased from Jackson ImmunoResearch (West Grove, PA, USA). Alexa Fluor 594-labeled donkey anti-goat IgG was purchased from Life Technologies (Carlsbad, CA, USA). 4′,6′-diamidino-2-phenylindole (DAPI) and Cy3-labeled goat anti-mouse IgG were purchased from Thermo Scientific (Waltham, MA, USA).

### Plasmid construction

Recombinant plasmids that express each NDV protein, were constructed by amplifying the coding regions from cDNA using PCR. PCR products were digested and ligated into p3 × Flag-CMV-14. The human GRP78 and GRP94 promoter luciferase reporters were constructed in pGL3-Basic as previously described^21^.

### NDV infection and NDV protein transfection

A549 cells (2 × 10^5^) per well in 6-well dishes were infected with NDV strains Herts/33 at a multiplicity of infection (MOI) of 1.0 and incubated at 37 ˚C for 1 h before complete medium was added. Infected cells were analyzed at 24 h post-infection (hpi). 2 μg of the plasmid were transfected into A549 cells and then harvested 24 h post-transfection for further experiments. For autophagy induction and ER stress induction experiments, cells grown to 60% confluence in 6-well cell culture plates were incubated in fresh medium containing rapamycin (100 nM) or thapsigargin (Tg; 300 nM) dimethyl sulfoxide (DMSO) was used as a negative control.

### Western blotting

A549 whole cell lysates were harvested and lysed in RIPA lysis buffer. Protein concentrations were determined using the bicinchoninic acid (BCA) assay. Equal amounts of total proteins were separated on SDS-PAGE gels. Protein bands were transferred onto nitrocellulose filter membranes (Millipore, Billerica, MA). Membranes were incubated with primary antibodies and appropriate secondary antibodies. The protein bands were detected using enhanced chemiluminescence detection kits (Thermo Scientific, Inc., Waltham, MA, USA).

### Small interfering RNA (siRNA) assays

RNA interference was used to knock down Beclin-1, PERK, or ATF6. A549 cells grown to 60 to 70% confluence in 6-well plates were transfected with 80 pmol siRNA using 3 μl Lipofectamine 2000 (Invitrogen) in Opti-MEM medium (Invitrogen). After 24 h, cells were then transfected with NDV NP- or P-expressing plasmids. After an additional 24 h, cells were harvested for further experiments. Scrambled siRNA were used as a negative control. The siRNA were designed and synthesized by Gene Pharma Company (Shanghai, China) with the following sequences: siPERK (sense, 5′-GGUUGGAGACUUUGGGUUAUU-3′; antisense, 5′- UAACCCAAAGUCUCCAACCUU-3′), siATF6 (sense, 5′-GAACAG GAUUCCAGGAGAAUU-3′; antisense, 5′- UUCUCCUGGAAUCCUGUUCUU-3′), siBeclin-1 (sense, 5′-CUGGAUAAGCUGAAGAAAAUU-3′; antisense, 5′- UUUUCUUCAGC UUAUCCAGUU-3′), non-targeting control siRNA (sense, 5′-UUCUCCGAACGUGUCACGUTT-3′; antisense, 5′-ACGUGACACGUU CGGAGAATT-3′). The silencing efficiency was measured by examining endogenous protein expression by Western blotting analysis.

### Luciferase assays

A549 cells were cultured in 24-well plates at a density of 1 × 10^5^ cells per well. The cells were co-transfected with 200 ng of NDV plasmids and 100 ng of firefly luciferase reporter promoter vectors pGL3-GRP78-luc or pGL3-GRP94-luc. To normalize for transfection efficiency, 10 ng of the constitutive Renilla luciferase reporter pRL-TK (Promega) was added to each transfection. After transfection for 48 h, cells were harvested to quantify the luciferase activity using the Dual-Luciferase Reporter Assay System (Promega).

### XBP1 mRNA Splicing by IRE1

Total RNA from A549 cells was isolated using TRIzol Reagent (Invitrogen). cDNA synthesis was performed using a SuperScript III kit (Invitrogen) and an oligo dT primer (Invitrogen). The XBP1 gene was amplified by RT-PCR with the forward primer 5′-AAACAGAGTAGCAGCGCAGACTGC-3′ and the reverse primer 5′-GGATCTCTAAGACTAGAGGCTTGGTG-3′. The PCR products were further digested with the restriction enzyme *Pst*I, and then separated on a 2% agarose gel. For internal control, the complementary DNA of the β-actin messenger RNA (mRNA) was also amplified using the forward primer 5′-TCCTGTGGCATCCACGAAACT-3′ and the reverse primer 5′-GAAGCATTTGCGGTGGACGAT-3′.

### Immunofluorescence staining

A549 cells grown to 60 to 70% confluence on coverslips were co-transfected with each NDV protein-expressing vector and with plasmid pEGFP-LC3 for 48 h. Cells were fixed in 4% paraformaldehyde for 15 min, permeabilized with 0.5% Triton X-100 for 10 min, and blocked with 3% bovine serum albumin (BSA) for 30 min. The cells were then stained with anti-Flag mAb, followed by staining with secondary antibodies conjugated to Alexa Fluor 594 (Life Technologies). Nuclei were stained with 4′, 6′-diamidino-2-phenylindole (DAPI), and the co-localization of each NDV protein with LC3 was visualized using a Nikon C1-si confocal fluorescence microscope (Nikon Instruments Inc., Melville, NY).

### Statistical analysis

The data were expressed as means + standard deviations (SD). Significance was determined with the two-tailed independent Student’s t test. A p-value of <0.05 was considered statistically significant.

## Results

### The NDV NP and P proteins trigger autophagy

**Figure 1:**
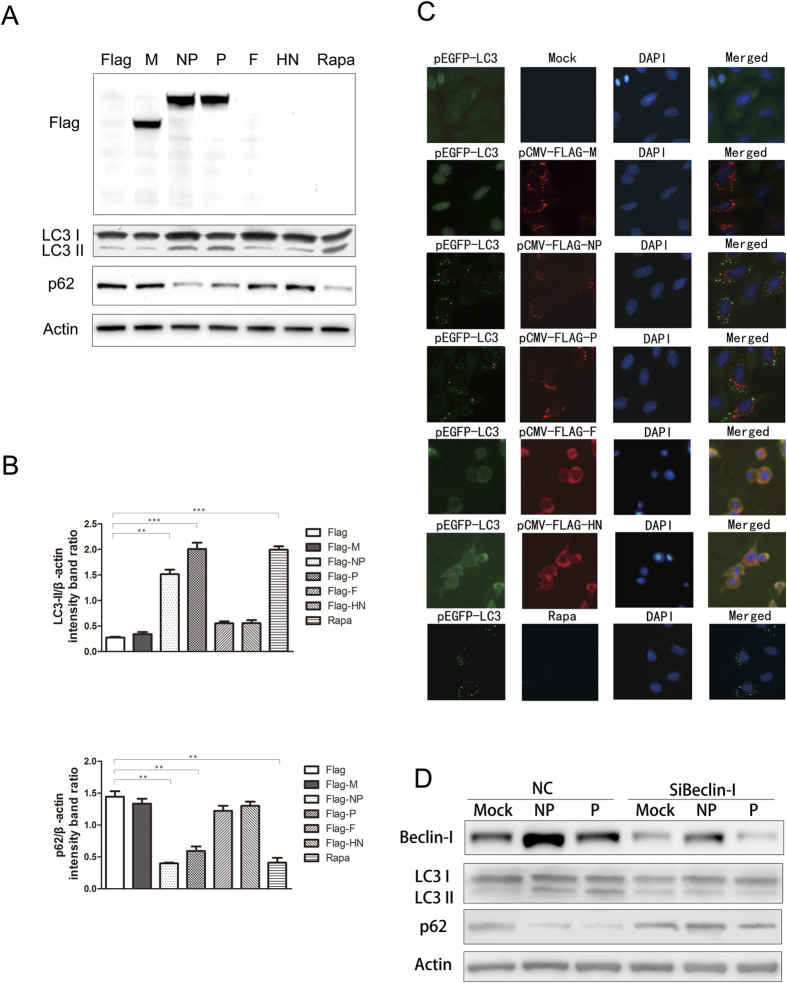
Multiple NDV proteins induce autophagy in A549 cells. (**A,B**) A549 cells were transfected with plasmids expressing FLAG-tagged NDV genes. Cells treated with rapamycin for 24 h were used as a positive control. At 24 h post-transfection, cells were harvested and western blotting was performed. The band intensities of LC3-II, p62 and β-actin were quantified, and the densitometric LC3-II/β-actin and p62/β-actin ratios from at least 3 independent experiments were calculated. (**C**) A549 cells were co-transfected with GFP-LC3 and FLAG-tagged NDV genes. At 24 h post-transfection, the cells were fixed and analyzed by using indirect immunofluorescence with anti-FLAG antibody. Green, GFP-LC3; red, FLAG-tagged NDV protein; blue, 4′,6′-diamidino-2-phenylindole staining. (**D**) A549 cells were transfected with a control siRNA or with siRNA directed against Beclin-1 (100 pmol/ml) for 24 h followed by NDV NP and P plasmid transfection. At 24 h post-transfection, cells were harvested for western blot analysis of LC3-II, p62, and β-actin.

Previous studies report that NDV can replicate and trigger autophagy in tumor cells^4^. To investigate whether individual NDV proteins can induce autophagy, we constructed corresponding recombinant plasmids with FLAG tags. Each FLAG-tagged NDV NP, P, M, HN, F, L expression plasmid was transfected into A549 cells and Western blotting was used to measure the conversion of LC3B-I to LC3B-II, and to determine p62 (SQTSM1) expression levels, as for p62 (SQTSM1) is an adaptor of LC3-II and is specifically degraded by the autophagy-lysosome pathway^33^. The NP, P, M proteins were detected using western blotting (Fig. 1A), while HN and F expression were only detectable using indirect immunofluorescence assays. The result of cells expressing L truncated protein was shown in supplementary data (Fig. S1). The densitometry ratio of LC3-II and β-actin in NP- or P-expressing cells was much higher than it was in the other NDV protein-expressing cells and mock-transfected A549 cells. Correspondingly, p62 was strongly degraded in NP- or P-transfected cells (Fig. 1A,B and Fig. S4), suggesting that the NDV NP and P proteins can induce autophagy in A549 cells. Cells treated with rapamycin were used as a positive control.

During autophagy, LC3 can redistribute from a diffuse cytoplasmic localization to a distinctive puncta cytoplasmic pattern. This process can be visualized by transfecting the cells with LC3 fused to the green fluorescent protein (GFP)^34^. Large amounts of punctate GFP-LC3 proteins were observed in NP- or P-expressing cells compared with mock-transfected A549 cells or cells transfected with M, HN or F expression plasmids (Fig. 1C). GFP-LC3 dot formation was also observed in positive control cells treated with an autophagy inducer, rapamycin.

To determine whether LC3 modification was caused by autophagic signaling rather than by NDV NP- or P-induced membrane alteration, we knocked down Beclin-1, which is critical to the signaling pathways that regulate autophagosome formation. Knockdown of Beclin-1 inhibited the conversion of LC3 and the degradation of p62 compared with the negative control groups, indicating that NDV NP and P can induce autophagy signaling in A549 cells (Fig. 1D).

### ER Stress is induced by NDV-infection and NP or P transfection

**Figure 2:**
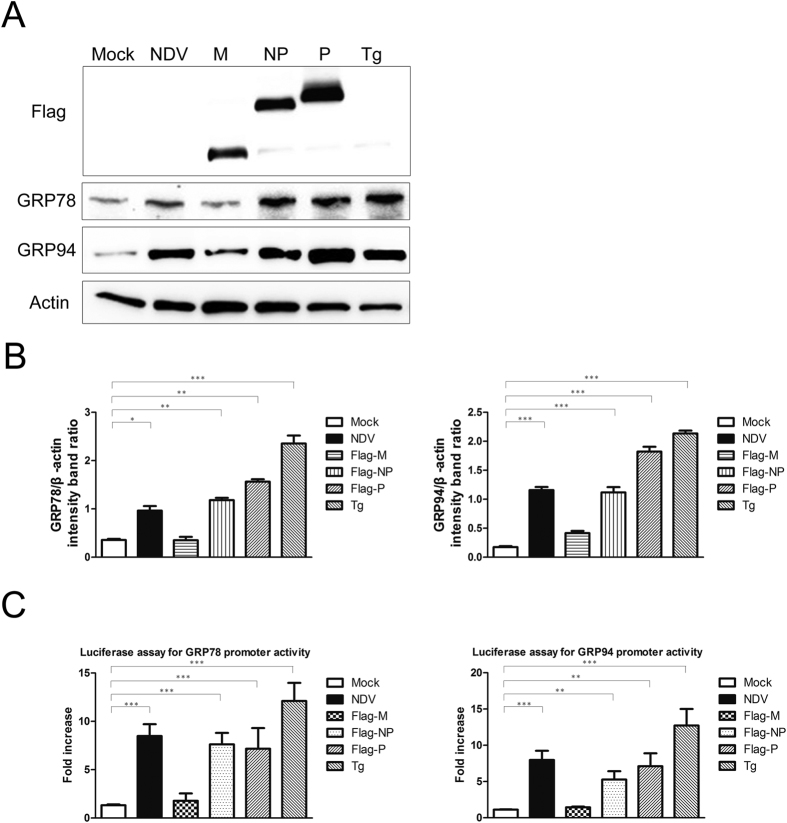
ER Stress is induced in NDV-infected or NDV P- or NP-expressing cells. (**A**) A549 cells were transfected with NDV NP, P or M plasmids for 48 h, infected with NDV at an MOI of 1, or treated with 300 nM Tg for 24 h were harvested for western blot analysis of GRP78, GRP94, β-actin as well as the NDV viral protein levels. (**B**) Band intensities of GRP78, GRP94, and β-actin were quantified, and the densitometric GRP78/β-actin and GRP94/β-actin ratios from at least 3 independent experiments are shown. (**C**) A549 cells were co-transfected with pGL3-GRP78-luc or pGL3-GRP94-luc and pCMV-FLAG-NP, pCMV-FLAG-P or pCMV-FLAG for 48 h, or infected with NDV at an MOI of 1, or treated with 300 nM Tg for 24 h and then harvested for quantification of firefly luciferase activities.

Several reports have shown that autophagy is induced via ER stress response^15^. Therefore we studied whether NDV could induce ER stress and subsequently regulate autophagy. To test this hypothesis, we assessed the expression of the ER stress marker proteins GRP78 and GRP94 in A549 cells after NDV infection, NP or P protein transfection, or Tg treatment as a positive control, M expression plasmids transfection were used as control which could not induce autophagy in A549 cells. For NDV-infected cells, GRP78 and GRP94 expression was strongly increased at 24 hpi when compared with uninfected cells (Fig. 2A,B). Substantial induction of GRP78 and GRP94 was also observed at 24 h post-transfection of NP or P, as compared with M-transfected cells (Fig. 2A,B and Fig. S4). The relative luciferase activities of the GRP78 and GRP94 promoters were significantly elevated in NP- or P -transfected cells and NDV-infected cells, but not in cells transfected with an empty vector or M expression plasmids (Fig. 2C). Taken together, these results demonstrate that the NDV NP and P proteins upregulate ER chaperones at both transcriptional and translational level.

### NDV induces ER stress via activation of UPR signaling through the PERK and ATF6 pathways

**Figure 3:**
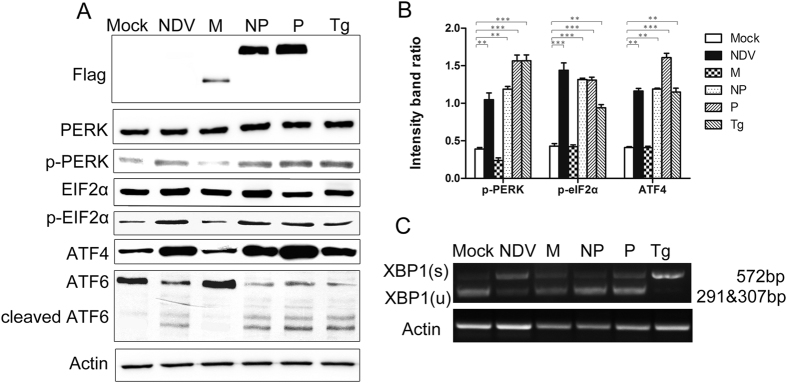
Activation of the UPR by NDV. (**A**) A549 cells were transfected with NDV NP or P plasmids, infected with NDV at an MOI of 1, or treated with 300 nM Tg for 24 h were harvested for Western blotting analysis of PERK, p-PERK, eIF2α, p-eIF2α, ATF4, ATF6, β-actin and NDV viral protein levels. (**B**) Densitometric p-PERK/β-actin, p-eIF2α/β-actin and ATF4/β-actin ratios from at least 3 independent experiments are shown. (**C**) Inactivation of the IRE1-XBP1 signaling pathway. Total cellular RNA was isolated from A549 cells and RT-PCR was performed using XBP1-specific primers. PCR products were digested with *Pst*I. Unspliced (u) XBP1 products, 291 bp and 307 bp; spliced (s) XBP1 products, 572 bp. Actin was used as the loading control.

To cope with ER stress, cells activate UPR via the sensor proteins, PERK, ATF6, and ERN1. We next examined which of them could be activated by NDV infection or by the expression of the NDV NP or P proteins. PERK and eIF2α were significantly phosphorylated in cells infected with NDV or transfected with either NP or P (Fig. 3A), while the total PERK and eIF2α protein remained unchanged. The abundance of the PERK and eIF2α effector ATF4 also increased, indicating that the PERK pathway was activated during NDV-induced ER stress.

When the ATF6 pathway is activated, ATF6 translocates from the ER to the Golgi apparatus and is cleaved by trans-membrane proteases, releasing the active N-terminal 50 kDa ATF6^35^. Both NDV infection and expression of the NDV NP or P protein increased the extent of degradation of the 90-kDa ATF6 precursor, yielding a 50 kDa cleavage product (Fig. 3A). These results indicate that NDV infection or protein NP and P transfection activates the ATF6 pathway of the UPR.

Finally, to determine whether the IRE1 pathway was also activated in NDV infection and NP or P expression, we analyzed the splicing of XBP1 mRNA. Activation of ERN1 by ER stress causes alternative splicing of XBP1 mRNA with the loss of a *Pst*I restriction site, while the unspliced XBP1 contains a *Pst*I site. In positive control cells treated with Tg, the spliced product was resistant to *Pst*I digestion (Fig. 3C). By contrast, the unspliced form of XBP1 mRNA (uXBP1) was mainly detected in cells infected with NDV as well as those overexpressing NDV NP or P protein. In addition, treatment with Tg significantly increased the level of phosphorylated IRE1 (p-IRE1), while p-IRE1 in cells infected with NDV or transfected with either NP or P remain unchanged (Fig. S3). Thus we concluded that XBP1 splicing is not induced by NDV and that the IRE1 pathway of the UPR was not activated.

### Induction of autophagosomes by NDV via ER Stress

**Figure 4:**
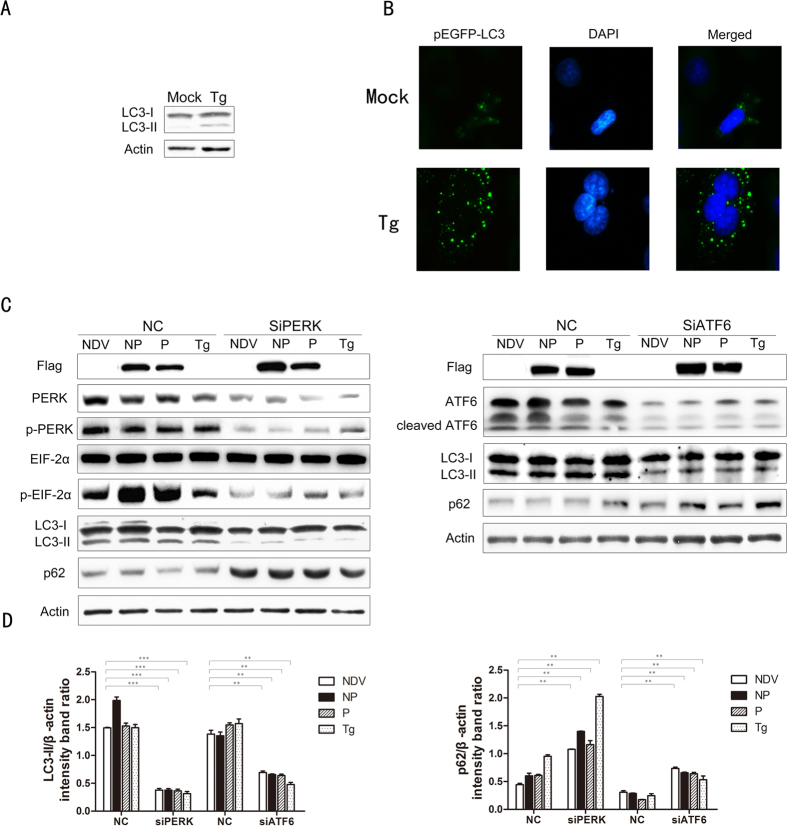
Induction of autophagy by NDV via the UPR. (**A**) A549 cells treated with 300 nM Tg for 24 h were analyzed by Western blotting for LC3 and actin. (**B**) A549 cells transfected with GFP-LC3 for 24 h were treated with 300 nM Tg and assessed by immunofluorescence analysis. (**C**) A549 cells transfected with a control siRNA or siRNAs directed against PERK or ATF6 (100 pmol/ml) for 24 h followed by NDV infection, or NDV NP and P protein expression plasmid transfection or Tg treatment. At 24 h post-transfection, cells were harvested and siPERK, siATF6, LC3-II, p62 and NDV viral protein NP or P expression were analyzed by western blotting. Cells treated with 300 nM Tg were used as a positive control. (**D**) Band intensities of LC3-II, p62, and β-actin were quantified, and the densitometric LC3-II/β-actin and p62/β-actin ratios from at least 3 independent experiments are shown.

Having shown that NDV can induce ER stress by activating the PERK and ATF6 pathways of the UPR, we next examined whether ER stress could induce autophagy in A549 cells. Treating cells with Tg significantly enhanced the conversion of LC3, as compared with mock-treated cells (Fig. 4A). Large amounts of punctate GFP-LC3 proteins were also observed in cells treated with Tg, indicating that ER stress can induce autophagy in A549 cells (Fig. 4B).

To detect whether NDV NP and P protein-induced autophagy via ER stress PERK or ATF6 was knocked down using siRNA and then LC3 conversion was evaluated in the context of NDV infection or after NP or P transfection. Knockdown of PERK reversed NDV or P and NP protein-induced LC3 conversion and p62 degradation (Fig. 4C). Similarly, in ATF6 knockdown cells, LC3 conversion and p62 degradation were also reduced (Fig. 4C). Taken together, these data indicate that the PERK and ATF6 pathways of the UPR are involved in NDV-induced autophagy.

### Inhibiting the UPR pathway suppresses NDV replication

**Figure 5:**
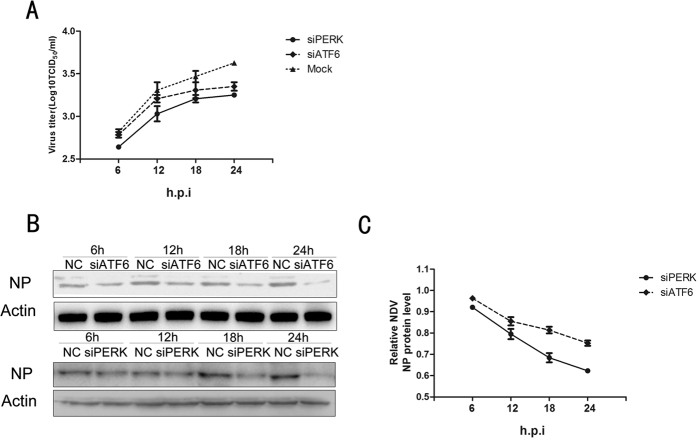
Effects of ER stress on NDV replication. A549 cells were transfected with a control siRNA or siRNAs directed against PERK or ATF6 (100 pmol/ml) for 48 h followed by NDV infection and harvested at the indicated times post-infection. (**A**) Virus titers from culture supernatants were determined and expressed as TCID_50_/ml. (**B**) NDV viral protein NP expression were analyzed by western blotting. (**C**) Quantification of NDV viral protein NP expression from Western blotting, normalized to expression in cells transfected with control siRNA.

We next determined the roles of the PERK and ATF6 pathways in NDV replication. NDV titers were significantly reduced in PERK and ATF6 knockdown cells, as compared with control cells (P < 0.01) (Fig. 5A). Consistent with this result, we also observed a reduced abundance of the NDV NP protein in PERK and ATF6 knockdown cells, as compared with control cells (Fig. 5B,C). In contrast with control cells, the amount of NDV particles in the cell culture supernatant of siPERK and siATF6 groups almost remained unchanged at 18 h and 24 h post infection (P > 0.05), although the abundance of the NP protein had decreased dramatically at the same time point (P < 0.01), possibly indicating that interfering with the UPR had a greater impact on reducing the ER protein folding capacity than on hampering virus assembly.

## Discussion

Virus-induced autophagy is an emerging area of investigation. Although autophagy is a cellular defense mechanism, several viruses have evolved strategies to use autophagic vesicles for their own replication. Individual viral proteins can directly modulate the induction of autophagy. For instance, the nonstructural proteins 2C and 3D are involved in autophagy induced by EMCV^21^. The nonstructural avian reovirus protein p17 triggers autophagy to enhance virus replication^36^. NS5B of hepatitis C virus has been reported to induce autophagy by interacting with autophagy protein ATG5^37^. Matrix protein 2 of influenza virus causes the accumulation of autophagosomes by blocking their fusion with lysosomes^38^. NDV induces autophagy in human tumor cells, as well as in chicken cells and tissues, but the specific NDV protein(s) involved in virus-induced autophagy were unknown^20^.

Here we demonstrated that the NP and P proteins are involved in NDV-induced autophagy, as shown both by immunofluorescence analysis of GFP-LC3 puncta formation and by Western blotting for LC3 conversion and for p62/SQSTM1 degradation, an index used to measure the extent of autophagy^33^.

Previous studies have shown that the class III PI3K/Beclin-1 pathway plays a crucial role in NDV-induced autophagy^12^. We found that knockdown of Beclin-1 reduced LC3 conversion and p62 degradation in NP- or P-transfected cells, indicating that the class III PI3K/Beclin-1 pathway plays an important role in NP- or P-induced autophagy.

It has been reported that some ER chaperones such as GRP78, GRP94 and calreticulin are activated in response to the ER stress^21^,^22^. In our study, we found that in cells infected NDV or cells transfected NDV NP and P, GRP78 and GRP94 were upregulated strongly at both the transcriptional and translational levels, suggesting that the NDV NP and P proteins are potent ER stress inducers to regulate the transcription of the ER chaperon against ER malfunction. Additionally, it has also been reported that GRP78 was required for stress-induced autophagy^39^. Whether GRP78 has any relevance with the NDV viral proteins during autophagy as well as activate ER stress remains to be explored in the future.

Prolonged ER stress in which misfolded/unfolded proteins exceed the capacity of the proteasome degradation system can trigger autophagy^40^. HCV induces the accumulation of autophagosomes via the induction of ER stress and all three arms of the UPR, but the HCV core protein only activates the PERK and ATF6 pathways^28^,^41^. We found that NDV infection and NP- or P-protein expression activated the PERK and ATF6 pathways, but not the ERN1-XBP1 pathway, and the autophagic process was inhibited in the PERK- or ATF6-knockdown cells to some extent. Although the NDV XBP(s) was more intense than the mock, M, NP or P samples, the p-IRE1 level was nearly the same, indicating the ERN1 pathway is not activated, and there may be other factors contributing to XBP1 splicing. Taken together, these results indicate that PERK and ATF6 pathways are specifically to trigger NDV-induced autophagy initiation, moreover, other signal pathways independent of ER stress may also regulate the NDV-induced autophagy as such process was not fully suppressed in the PERK- or ATF6-knockdown cells.

These results indicate that PERK and ATF6 pathways are specifically to trigger NDV-induced autophagy initiation, moreover, other signal pathways independent of ER stress may also regulate the NDV-induced autophagy as such process was not fully suppressed in the PERK- or ATF6-knockdown cells.

During virus infection, the production of viral proteins can activate PKR and/or PERK, leading to translation attenuation. Several viruses activate the ATF6 pathway to facilitate their replication, including WNV, HCV, and African swine fever virus (ASFV). In WNV-infected cells, ATF6 activation promotes WNV replication by suppressing signal transducer and late-phase IFN signaling^42^. Under ER stress, ERN-XBP1 activation can lead to JNK pathway induction, which is required for autophagosome formation^43^. ERN pathway inhibitor diminished virus yield significantly, which suggests that activation of ERN is essential for HCV infection and autophagy induction^44^. Whether such pathways have a role in NDV replication remain unknown.

In conclusion, our studies demonstrate that the NDV NP and P proteins are involved in NDV-induced autophagy and that this process is at least partially regulated by ER stress. Our results also suggest that autophagy induction involves two arms of the UPR, and interference of these two arms can suppress NDV replication. These data lay the foundation for future studies to understand better the molecular mechanisms of autophagy in relation to NDV infection.

## Additional Information

**How to cite this article**: Cheng, J.-H. *et al.* Newcastle disease virus NP and P proteins induce autophagy via the endoplasmic reticulum stress-related unfolded protein response. *Sci. Rep.* **6**, 24721; doi: 10.1038/srep24721 (2016).

## Supplementary Material

Supplementary Information
