# Supplementary material for: Newcastle disease virus NP and P proteins induce autophagy via the endoplasmic reticulum stress-related unfolded protein response
Source: Sci Rep. 2016 Apr 21;6:24721. doi: 10.1038/srep24721 (PMC4838823; doi:10.1038/srep24721)
Supplement: Supplementary Information [file srep24721-s1.pdf]

Newcastle disease virus NP and P proteins induce autophagy via the endoplasmic reticulum stress-related unfolded protein response

Jing-Hua Cheng[1, 2], Ying-Jie Sun[1], Fan-Qing Zhang[1, 3], Xiao-Rong Zhang[2], Xv-Sheng Qiu[1], Li-Ping Yu[2], Yantao Wu[2, *], Chan Ding[1, *]

Supplementary Figures

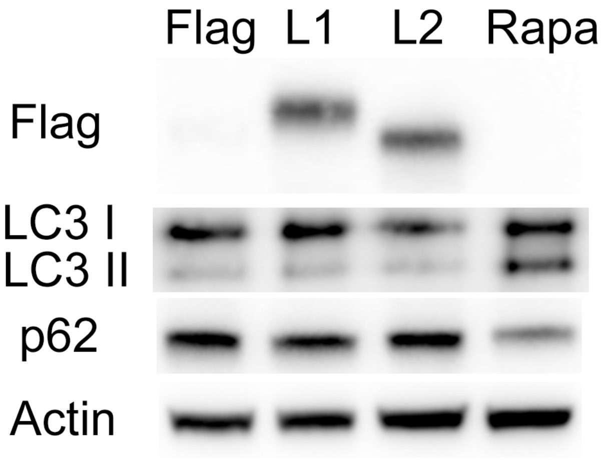

Figure S1. Separate L protein could not induce autophagy in A549 cells. A549 cells were transfected with two Separate L-expressing plasmids. Cells treated with rapamycin for 24 h were used as a positive control. At 24 h post-transfection, cells were harvested and LC3-II, p62 and β-actin were analyzed by western blotting.

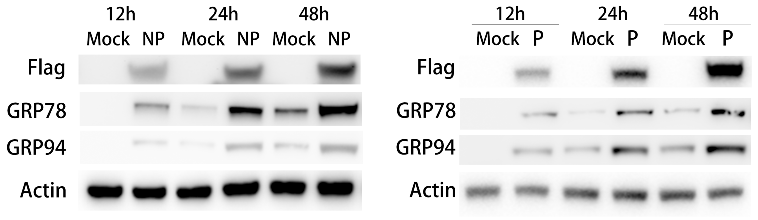

Figure S2.ER Stress is induced in NDV-infected or NDV NP- or P-expressing cells from 12 to 48 h. A549 cells were transfected with NDV P- or NP- plasmids for 12, 24, and 48 h, The GRP78, GRP94, β-actin as well as the NDV viral protein levels were analyzed by western blot assay at indicated time points post-transfection.

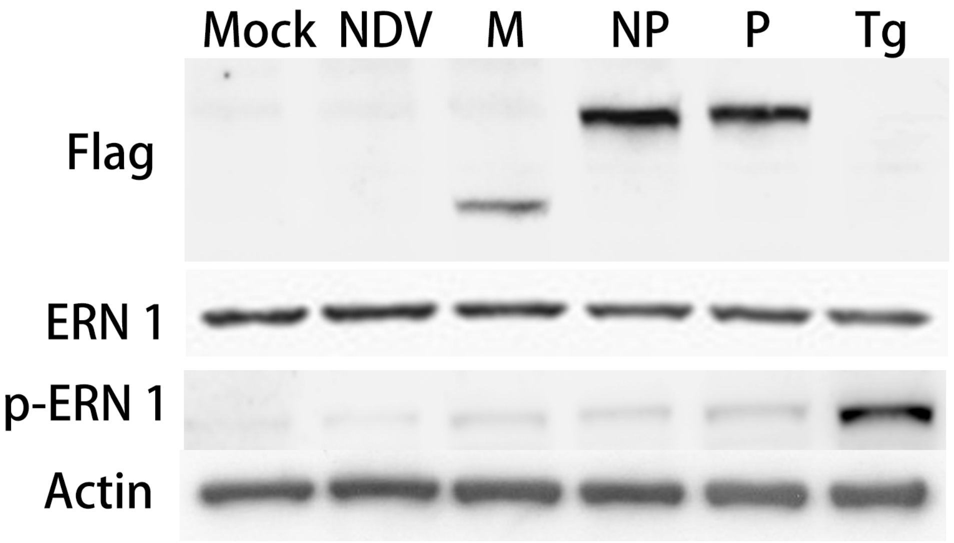

Figure S3. IRE 1 pathway was slightly activated during NDV infection but not NDV protein transfection. A549 cells were transfected with NDV NP, P or M plasmids, infected with NDV at an MOI of 1, or treated with 300 nM Tg for 24 h were harvested for Western blotting analysis of ERN1, p-ERN1, β-actin and NDV viral protein levels

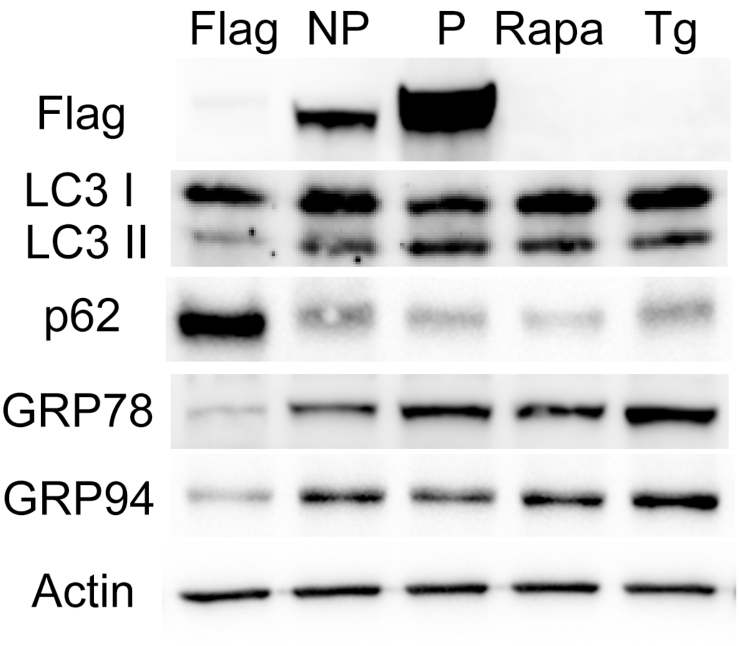

Figure S4. Autophagy and ER Stress is induced by NP or P protein from the lentogenic strain. A549 cells were transfected with NDV NP- or P- plasmids from lentogenic strain for 24 h. The LC3-II, p62, GRP78, GRP94 and β-actin were analyzed by western blot assay.
